# Multimodal Intraoperative Neurophysiological Monitoring in *TUBB4A*‐Related Hypomyelinating Leukodystrophy During Scoliosis Correction: A Case Report

**DOI:** 10.1155/crnm/8210514

**Published:** 2026-11-14

**Authors:** Marta Rodrigues de Carvalho, Eduardo Henrique Chiovato Abdala, Carlos Fernando Pereira da Silva Herrero, Narcélio Mendes Ferreira Filho, Vitor Marques Caldas

**Affiliations:** ^1^ Clinical Neurology and Neurophysiology Sector Base Hospital of the Federal District Brasilia Brazil; ^2^ Orthopedics and Traumatology Sector Paranoá Regional Hospital Brasilia Brazil; ^3^ Department of Orthopedics and Anesthesiology University of São Paulo Ribeirão Preto Brazil usp.br; ^4^ Orthopedics Department Spine Institute Brasilia Brazil; ^5^ Intraoperative Neurophysiology Sector Focal Clinic Brasilia Brazil

**Keywords:** evoked potentials, hypomyelinating, intraoperative monitoring, leukodystrophy, scoliosis, TUBB4A protein, with atrophy of the basal ganglia and cerebellum

## Abstract

Intraoperative neurophysiological monitoring (IONM) is essential in spinal deformity surgery; however, it may be limited in patients with hypomyelinating leukodystrophy and impaired long‐tract conduction. Data on the neurophysiological features of leukodystrophies are scarce. Therefore, we report a case of a 15‐year‐old patient with hypomyelinating leukodystrophy who underwent surgical correction of severe scoliosis (Cobb angle 76°), with a surgical duration of 360 min. During the procedure, transcranial motor evoked potentials (MEPs) and somatosensory‐evoked potentials (SSEPs) could not be obtained despite maximal stimulation parameters, a finding attributed to severe central hypomyelination. In contrast, the bulbocavernosus reflex (BCR) remained reproducible and stable throughout, documenting the integrity of the segmental sacral reflex circuit (S2–S4) and its peripheral innervation. Surveillance was complemented by free‐running electromyography (EMG) and surgeon feedback. The patient had no postoperative neurological deficit, with maintained motor and sphincter function. In patients with hypomyelinating leukodystrophy, the absence of MEPs and SSEPs may not indicate surgical injury but rather reflect impaired long‐tract conduction. The preserved BCR offers a valuable segmental monitoring modality, and a concomitant multimodal strategy—BCR, free‐running EMG, and surgeon feedback—should be considered when evoked potentials are unobtainable. This is the first report detailing intraoperative MEPs and SSEPs findings in this rare population, underscoring the need for adapted IONM protocols in patients with rare and complex neurological comorbidities.

## 1. Introduction

Hypomyelinating leukodystrophies (HLDs) comprise a heterogeneous group of inherited disorders that primarily affect central nervous system (CNS) white matter [1]. Recent advances—especially in genomics, neuroimaging, and pathology—have reshaped definitions, expanded the catalog of disorders, and improved diagnostic specificity [2]. The concept of leukodystrophies has evolved over the past century. An early familial white matter disorder, now known as Pelizaeus–Merzbacher disease, was first described over 100 years ago. The term “leukodystrophy” was defined in 1928 as a hereditary, progressive degeneration of CNS white matter, and refined in 1984 to emphasize genetic disorders primarily affecting myelin or oligodendroglia with progressive functional decline. Now, it has been broadened to encompass any heritable disorder that primarily targets CNS white matter, regardless of peripheral nervous system involvement [2]. Over the past decade, the catalog of HLDs has expanded substantially, with more than 20 specific genes reported, reflecting major progress in gene discovery [3].

Data on the neurophysiological features of leukodystrophies are scarce. Previous studies conducted in outpatient settings indicate that patients with HLDs have prolonged conduction velocities and absent motor‐evoked potentials (MEPs) or somatosensory‐evoked potentials (SSEPs) [4–6]. A systematic literature search using the MeSH terms (“Intraoperative Neurophysiological Monitoring”[Mesh]) AND “Leukodystrophy, Hypomyelinating, 6” [Supplementary Concept]; (“Monitoring, Intraoperative”[Mesh]) AND “Pelizaeus‐Merzbacher Disease”[Mesh]; (“Leukodystrophy, Hypomyelinating, 6” [Supplementary Concept]) AND “Surgical Procedures, Operative”[Mesh]; (“Leukodystrophy, Hypomyelinating, 6” [Supplementary Concept]) AND “Anesthesia”[Mesh]; (“Leukodystrophy, Hypomyelinating, 6” [Supplementary Concept]) AND “Perioperative Period”[Mesh]; and (“Leukodystrophy, Hypomyelinating, 6” [Supplementary Concept]) AND “Perioperative Care”[Mesh] confirms a significant gap in the literature regarding intraoperative monitoring in patients with HLDs.

To the best of our knowledge, this is the first reported case of intraoperative neuromonitoring (IONM) in genetically confirmed *TUBB4A* leukodystrophy. Therefore, in this report, we aimed to describe the importance and particularities of neurophysiological monitoring in a patient with a rare case of HLD, highlighting technical parameters, preoperative evaluation, and postoperative outcomes.

## 2. Case Presentation

A 15‐year‐old male patient presented with a history of delayed neuropsychomotor development and loss of previously‐acquired motor and cognitive milestones. Presurgical physical examination data included a cognitive assessment using the Wechsler Intelligence Scale for Children (WISC‐IV), which classified the patient with an intellectual disability. This hindered the neurological physical examination due to difficulties in comprehension and following commands; however, notable findings included the patient being nonambulatory and presenting with severe tetraparesis, with strength graded as I in the lower limbs and III in the upper limbs on the Medical Research Council (MRC) scale. No response to vibratory stimuli was observed in the lower limbs. Cranial nerve examination revealed uncoordinated eye movements. The patient exhibited globally hyperactive reflexes. Spasticity was classified as Grade 3 on the Modified Ashworth Scale (considerable increase in muscle tone with difficulty in passive movement), and his gross motor skills were classified as Level V on the gross motor function classification system (GMFCS). The patient maintained urinary and fecal continence, requiring no external maneuvers for bowel or bladder emptying. The protection of these functions was one of the main concerns of the surgical team.

Extensive investigation had previously been carried out. Magnetic resonance imaging (MRI) of the brain revealed a diffuse increase in signal intensity within the deep and subcortical white matter of the cerebral hemispheres on T1‐weighted sequences, with relatively preserved signal intensity. A similar appearance, with reduced signal intensity, was observed involving the dentate nuclei and the dorsal portion of the pons. The corpus callosum showed mild thinning. Prominence of the cerebellar folia was noted, particularly in the upper thirds. Diffusion‐weighted images showed no evidence of restricted water molecule diffusion (Figure 1). Cervical MRI showed preserved morphology and signal of the spinal cord, without atrophy or focal lesions. Thoracic MRI showed a scoliotic curvature at the thoracolumbar junction, convex to the left; left paracentral disc protrusions at T2‐T3 and T3‐T4, causing dural indentation but no nerve root compression; and a spinal cord with preserved morphology and signal, without focal lesions or atrophy. Lumbosacral MRI showed a scoliotic curvature at the thoracolumbar junction, convex to the left; preserved dimensions of the spinal canal and intervertebral foramina; a conus medullaris with normal shape and signal at the thoracolumbar junction; and cauda equina nerve roots with normal anatomical distribution within the dural sac.

**FIGURE 1:**
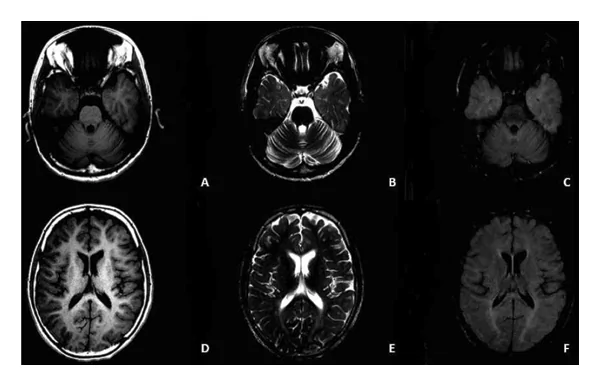
Brain MRI. (A–C) Axial T1, T2, and 2/FLAIR: emphasis is placed on the temporal pole and cerebellum. (D–F) Basal ganglia, corpus callosum, and subcortical white matter.

Previous outpatient SSEP examination of the lower limbs (tibial nerves) 5 years past the surgery were performed using electrical stimulation with recording over the lumbar, thoracic, and paramedian centroparietal cortical (CPz) regions, referenced to frontopolar (Fpz). The N20, P30, and P37/N45 waves appeared poorly defined, with prolonged absolute and interpeak latencies. Auditory‐evoked potentials with short latency were obtained 5 years ago via monaural stimulation using rarefaction‐type clicks with contralateral masking, an intensity of 80 dB, and a frequency of 10.11 Hz; recording at ear lobes (left‐A1 and right‐A2) according to the 10–20 system, with the reference at central cortical region (Cz) evidenced poorly elaborated and poorly reproducible waves. There were no results for upper‐limb SSEPs (median or ulnar nerves). Electroneuromyography performed 10 years ago showed no abnormalities. No recent follow‐up or perioperative neurophysiological tests were performed. Several genetic tests were performed, with a multigene next‐generation sequencing panel for dystonia identifying a heterozygous pathogenic variant in the *TUBB4A* gene (NM_006087.2 c.1172G > Ap(.Arg391His).

The patient developed a severe scoliotic deformity with 76° pelvic obliquity, which resulted in pressure ulcers, difficulty with personal hygiene, and significant pain. Therefore, the spine surgery team elected to perform a corrective procedure including multiple osteotomies (resection of the facets, ligamentum flavum, and laminae) aimed at reducing stiffness and allowing translational and rotational correction via segmental distraction and compression from T2 to S1 (Figure 2). Because of the magnitude and neurologic risk of the procedure, IONM was performed. Our protocol included transcranial electrical MEPs, SSEPs, free‐running and triggered electromyography (EMG), and continuous assessment of the bulbocavernosus reflex (BCR) (Figures 3, 4, 5).

**FIGURE 2:**
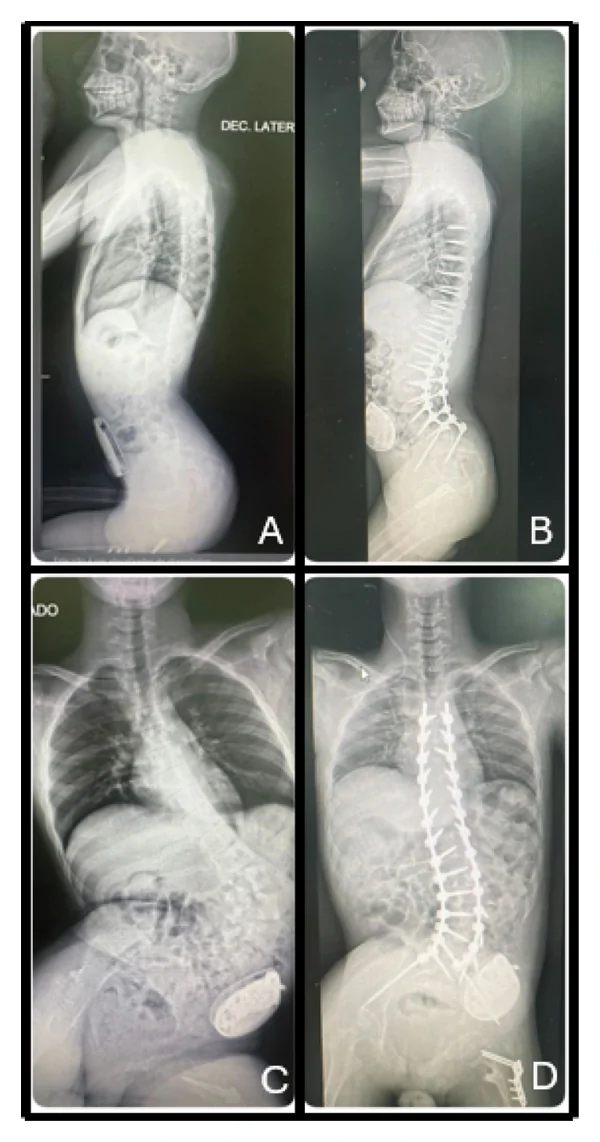
Pre‐ and postoperative X‐rays. (A) Preoperative profile incidence. (B) Postoperative profile. (C) Preoperative anteroposterior incidence. (D) Postoperative anteroposterior incidence.

**FIGURE 3:**
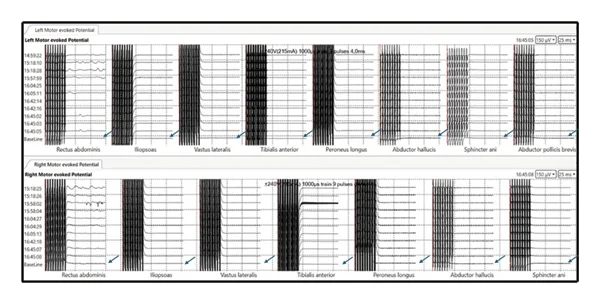
Motor‐evoked potentials performed throughout the surgical procedure. The figure shows the absence of reproducible responses despite maximal stimuli in the bilateral rectus abdominis, iliopsoas, vastus lateralis, tibialis anterior, peroneus longus, abductor hallucis, and sphincter ani, and left abductor pollicis brevis. Stimulation: Train of 9 stimuli, interstimulus interval 4 ms, 215 mA, 1 ms duration. Sensitivity 150 μV/mm. Arrows: The blue arrows point to the flat trace of an absent motor‐evoked potential.

**FIGURE 4:**
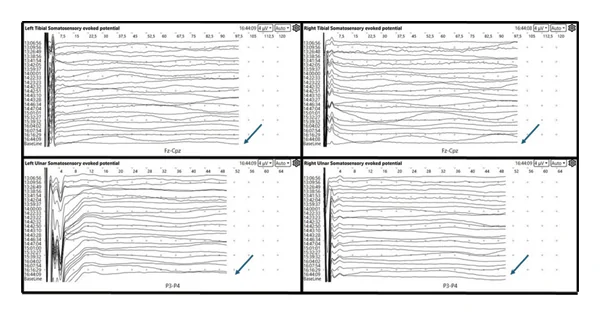
Somatosensory‐evoked potentials performed throughout the surgical procedure. The figure shows the absence of reproducible responses despite maximal stimuli in the (A, B) right and left ulnar and (C, D) right and left tibia. Stimulation: up to 65 mA, pulse duration of up to 1 ms, and repetition rate of 2.79–3.11 Hz. Arrows: The blue arrows point to the nonreproducible somatosensory‐evoked potential responses.

**FIGURE 5:**
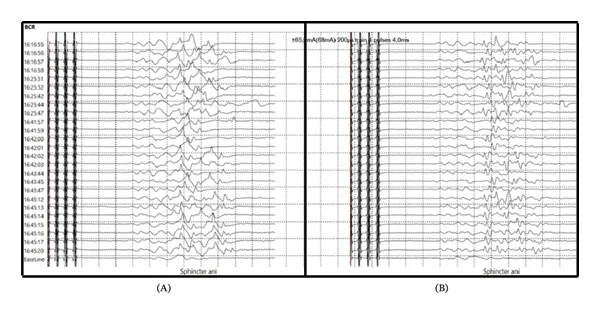
Bulbocavernous reflex performed throughout the surgical procedure. The figure shows the presence of stable and reproducible responses (train of 2–4 stimuli, interstimulus interval 2 ms, 65 mA). (A) Left sphincter ani. (B) Right sphincter ani.

During the procedure, total intravenous anesthesia using 2% propofol (6 mg/kg/h) and remifentanil (0.1 μg/kg/h) was administered. No other adjuvant medications were used, including ketamine, dexmedetomidine, volatile anesthetics, or lidocaine. A small dose of rocuronium was given for intubation, which was pharmacologically reverted by sugammadex. The patients’ temperature was maintained at adequate levels (35°C), and the mean arterial pressure was kept between 55 and 80 mmHg during the whole procedure. There was no report of the use of vasopressor drugs. The depth of anesthesia was monitored by electroencephalography (EEG) in order to adjust and avoid levels of attenuation/suppression of baseline brain activity that could interfere with MEPs. The level of curarization was also assessed using the train‐of‐four (TOF) technique in order to ensure that residual muscle relaxation did not iatrogenically suppress MEPs. Applying this technique resulted in responses exceeding 90% (0.9) prior to the recording of MEPs.

The laboratory results immediately prior to the surgery included hemoglobin 14.0 g/dL; hematocrit 42%; arterial blood gas pH 7.39, pO_2_ 218 mmHg, and pCO_2_ 39 mmHg; and HCO_3_ 24 mmOL/L. Intraoperative laboratory results included hemoglobin 12.6 g/dL; hematocrit 38%; arterial blood gas pH 7.36, pO_2_ 183 mmHg, and pCO_2_ 38 mmHg; and HCO_3_ 22 mmOL/L. Postoperative results included hemoglobin 11.4 g/dL; hematocrit 34%; arterial blood gas pH 7.21, pO_2_ 165 mmHg, and pCO_2_ 46 mmHg; and HCO_3_ 18 mmOL/L. The surgery lasted approximately 6 h, with blood loss estimated at 900 mL. During the surgery, one unit of packed red blood cells was transfused, with another unit administered after the procedure. No episodes of hypotension were reported.

All IONM was performed with a 16 channel IONM software by Neuro‐IOM (NeurosoftLtd., Ivanovo, Russia). For MEPs cortical stimulation, corkscrew‐stimulating electrodes were positioned on the scalp at C3 and C4, strictly adhering to the international 10–20 system. This configuration aimed to optimize motor cortex activation, ensuring targeted and reproducible stimulation. The montage used for MEPs stimulation was central (C3‐left and C4‐right) delivered through biphasic pulses. Transcranial stimulation was delivered through trains consisting of 5–9 pulses (maximal of nine pulses) using a constant voltage stimulator, with a maximal current of 215 mA. Each pulse had a duration of 1 millisecond, with an interstimulus interval of 4 milliseconds. The bandpass filter was 100–1500 Hz. MEPs recordings were performed regularly between successive trains to prevent neuronal fatigue and ensure cortical excitability recovery. MEPs were recorded using needle recording electrodes placed bilaterally in the rectus abdominis, iliopsoas, vastus lateralis, tibialis anterior, peroneus longus, abductor hallucis, and sphincter ani, and left abductor pollicis brevis. Alert criteria were established as a significant amplitude drop of more than 50% relative to the baseline or a complete loss of signal. As we did not obtain reproducible results from the beginning of the procedure, this response pattern was also defined as an alert criterion, and we immediately notified the surgical team.

Cortical SSEPS were recorded using the system’s bandpass filter from 100 to 3000 Hz using parietal (left‐P3 and right‐P4), frontal, and centroparietal midline (Fz/Cpz) electrodes according to the international 10–20 system. Ulnar and tibial stimuli were applied with an intensity of up to 65 mA, a pulse duration of 200 microsecond to 1 millisecond, a repetition rate of 2.79–3.11 Hz, and a band pass filter of 30–3000 Hz. A minimum averaging rate of 200 replications was used. However, reproducible responses with typical morphologies and latencies for this age group were not obtained.

BCR stimulation of the pudendal nerve was achieved with pad electrodes placed at the dorsal base of the shaft of the penis (intensity: 20–65 mA; train: 2–4 stimuli; interstimulus interval: 2 ms). BCR recording was achieved with needle electrodes inserted in bilateral sphincter ani muscles. Recording was performed using 30–1000 Hz filters and a gain of 50–100 μV/division. Reproducibility was confirmed by 10 superimposable responses in consecutive traces. A polyphasic response with a latency of around 35 milliseconds was obtained, remaining stable throughout the whole surgical procedure. Alert criteria were established as an amplitude drop of more than 50% relative to the baseline or a complete loss of signal.

Spontaneous (free‐running) EMG was continuously recorded bilaterally from the rectus abdominis, iliopsoas, vastus lateralis, tibialis anterior, peroneus longus, abductor hallucis, and sphincter ani, and left abductor pollicis brevis using filters of 30–1000 Hz and a gain of 50–100 μV/division. Alarm signals included continuous neurotonic discharges and bursts of Type B and C discharges. Assessment of the medial integrity of pedicle screws was performed using monopolar electrical stimulation, with the cathode connected to the screw (via a connector) and the anode placed as a reference needle in muscle tissue, using a current ranging from 0 to 20 mA with 1 mA increments. Recordings were obtained from the muscles of the corresponding myotome, using filters set at 30–1000 Hz and a gain of 50–100 μV/division. The protocol varied according to the vertebral level, accounting for anatomical and physiological differences between the lumbar and thoracic spine. In the lumbar spine, single pulses with a duration of 200 µs were used. Screws with a stimulation threshold ≥ 10 mA were considered safe. Thresholds of 6–10 mA were interpreted as indicating a possible medial breach or thin wall, prompting inspection or revision, and thresholds < 6 mA were considered indicative of a definite medial breach with nerve root contact, mandating screw revision. In the thoracic spine, a four‐pulse train was used, with an interstimulus interval of 2 ms, pulse duration of 0.2 ms, frequency of 3 Hz, and intensity of 10–15 mA. Screws with a stimulation threshold ≥ 10 mA were considered safe.

Postoperative motor deficit was defined as a reduction of at least one grade in muscle strength, as assessed by the MRC Scale for muscle strength. Patients’ neurological function was systematically examined, searching for early detection of any neurological changes (defined as weakness, changes in sensation, and sphincter dysfunction). No deficits were identified in the immediate or late postoperative period in the aforementioned domains. Postoperative thoracolumbar spine CT taken 2 weeks after the surgery showed extensive thoracolumbar arthrodesis, from T4 to the ilia; the right screws at T4 and L5 followed a course within the lateral recess; the left screws at T5 and from T7 to T10 were laterally displaced (extrapedicular) adjacent to the costovertebral joint; and surgical decompression was present at multiple posterior arches. A large soft‐tissue collection in the thoracolumbar region—difficult to fully characterize via this modality—apparently extends from T10 to L3, measuring 11.7 × 4.0 × 7.4 cm, with an estimated volume of approximately 180 mL. A postoperative MRI was not performed. The patient developed a surgical wound infection and required hospitalization for 6 weeks to receive broad‐spectrum antibiotics. At the 4‐month postoperative reassessment in the outpatient clinic, the patient maintained the same preoperative neurological status.

## 3. Discussion

Myelin is a multilayered sheath with insulating properties produced by oligodendrocytes in the CNS. It is involved in the efficient transmission of nerve impulses and energy‐metabolic support [7, 8]. Within the context of HLDs, myelin production is compromised by numerous genetic alterations that culminate in several pathophysiological mechanisms, including dysfunction of intracellular structures such as the endoplasmic reticulum, components of the multi‐tRNA synthetase complex, and mitochondrial apoptosis [3].

Hypomyelination with atrophy of the basal ganglia and cerebellum (H‐ABC) or hypomyelinating leukodystrophy Type 6 (HLD6) was first described in 2002 (OMIM 612438) [9]. In 2013, only 22 cases had been reported and a *de novo* mutation in the *TUBB4A* gene was identified in all affected individuals, indicating that it may be the genetic cause of the disease [10]. *TUBB4A* comprises the β‐tubulin protein family, widely expressed in the CNS, and forms heterodimers with α‐tubulins, forming microtubules essential for the cytoskeleton [11]. Its role in the pathophysiology of HLD may be due to alterations in neuronal migration, differentiation, and axonal orientation and maintenance [10]. *TUBB4A* is known to be located primarily in mature myelinating neurons and oligodendrocytes, particularly in the cerebellum, white matter tracts, and striatum [12].

The clinical presentation is generally heterogeneous, with varying degrees of motor delay, intellectual disability, pyramidal signs, extrapyramidal symptoms, cerebellar ataxia, bulbar dysfunction, gait abnormalities, speech disorder, and oculomotor abnormalities [1, 13]. A recent study of over 200 patients with childhood‐onset *TUBB4A*‐related leukodystrophy showed that scoliosis is one of the most common medical complications, with patients in the early childhood‐onset form being more severely affected [14].

Myelin dysfunction in leukodystrophies directly affects the physiological mechanisms underlying evoked potentials, resulting in dysfunctions that include prolonged latencies, altered amplitudes and morphologies, or the absence of responses, with studies primarily evaluating somatosensory, auditory, and visual evoked potentials [15–19]. The literature on *TUBB4A* is limited and does not address surgical procedures or neuromonitoring. Few studies report the findings from neurophysiological studies in patients with *TUBB4A*‐related HLD; hence, the present study may be the first to report the findings from IONM. One study reported the findings from a human patient and animal models with this diagnosis, showing an absence of Waves III–V in the human model and altered latencies of Waves III and V in the animal model [20]. Another study comparing SSEPs and MEPs responses in human and animal models showed delayed SSEPs latencies in the upper limbs and their absence in the lower limbs in the human patient and delayed response to SSEPs and direct and indirect fused wave response MEPs in the animal model. The same study using animal models demonstrated that corticospinal conduction velocities were significantly reduced, by more than three‐to‐fourfold, compared with the normal model [21]. Finally, a relatively recent study describing a novel *TUBB4A* mutation reported that the patient presented with delayed responses of visual‐evoked and SSEPs [22].

The absence of transcranial MEPs observed in this case is physiologically consistent with a diagnosis of HLD, reflecting a critical failure in rapid, synchronized conduction along the corticospinal tract. The process of demyelination impairs the synchrony of action potentials. Animal models have demonstrated asynchronous transmission of action potentials and corresponding postsynaptic currents in the neonatal spinal cord and in *in vitro* hippocampal preparations [22]. The underlying mechanism lies in severe central hypomyelination, which precludes saltatory conduction and necessitates continuous, inefficient conduction. This phenomenon results in marked temporal dispersion of the descending volley, causing action potentials to arrive at the spinal cord’s anterior horn in a desynchronized manner [23]. Given that the corticospinal tract is the primary anatomical substrate for voluntary movement, relying on large‐caliber, densely myelinated axons to propagate D (direct) and I (indirect) waves, any compromise to the myelin sheath degrades motor signal quality [23, 24]. The pathophysiology behind the absence of a muscle response in MEPs stems from impaired temporal summation at the alpha motor neuron level. For motor neuron firing and subsequent muscle contraction to occur, D and I waves must reach the synapse within an extremely brief interval to reach the depolarization threshold. In cases of hypomyelination, the attenuation and delay of these waves prevent effective summation [23]. Furthermore, altered cortical excitability, inherent to white matter pathologies, hinders the initial generation of I‐waves, a process exacerbated by anesthetic effects. Agents such as propofol and inhalational anesthetics (even at low concentrations) preferentially suppress I‐wave generation, narrowing the margin for eliciting responses in patients with marginal conduction reserve [25]. Chronic structural factors also contribute to this electrophysiological silence. Chronic axonal degeneration secondary to prolonged hypomyelination reduces the total pool of recruitable motor fibers, thereby diminishing the potential signal amplitude [24, 26]. In a surgical context, the presence of long‐standing scoliosis presents additional challenges; chronic mechanical deformity can cause spinal cord stretching or microvascular compression, and the observation of absent or severely degraded baseline signals is common in cases of neuromuscular scoliosis, even with optimal technical execution [27].

In contrast to the disruption of long pathways, the preservation of the BCR throughout the procedure highlights the integrity of the segmental sacral reflex circuit. The BCR is mediated by an olygosynaptic arc involving Segments S2–S4 and Onuf’s nucleus in the sacral spinal cord [28]. The maintenance of this reflex, despite central pathology, is explained by the short length of the pathway and the preservation of peripheral myelin. While MEPs rely on long tracts extensively affected by leukodystrophy, the BCR traverses a short anatomical distance; hence, this minimizes the impact of temporal dispersion and allows the olygosynaptic pathway to reach the response threshold [29]. Furthermore, the integrity of the pudendal nerve is a determining factor. In central leukodystrophies, as in the reported case, myelin produced by Schwann cells in the peripheral nervous system is typically spared, ensuring that the afferent and efferent limbs of the reflex arc remain functional [12]. This finding suggests relative sparing of sacral spinal cord pathways and their local reflex circuits compared with rostral projection tracts [29, 30]. However, it is imperative to emphasize that BCR preservation does not equate to the integrity of long motor or sensory tracts; the BCR documents only the functionality of the sacral segmental arc and does not serve as an indicator of the preservation of corticospinal or lemniscal pathways above the sacral level.

The absence of long tract baseline responses in a patient with HLD makes it difficult to distinguish between disease‐related signal loss and acute surgical injury, representing a fundamental limitation of monitoring initiated without an obtainable baseline. Without a baseline signal, the monitor loses the ability to detect relative deterioration, and electrophysiological silence ceases to function as an alarm [27]. This redefines the utility of MEPs or SSEPs: when present, even with reduced amplitude and prolonged latency, they serve as a valuable reference; when absent from the outset, they shift the burden of surveillance to complementary modalities. In this context, multimodal monitoring plays a central role. Free‐running EMG offers continuous surveillance for nerve root and spinal cord irritation, independent of long‐tract integrity, by detecting pathologic discharges that signal impending injury. Direct feedback from the surgeon regarding intraoperative steps integrates with the monitoring system as a qualitative source of information [31, 32]. Finally, the wake‐up test, while limited in application for patients with baseline motor impairment, remains an alternative when electrophysiological modalities yield uninterpretable results. However, its feasibility must be discussed with the team beforehand, weighing the benefits of clinical confirmation against the risks associated with intraoperative awakening [27, 33].

These monitoring challenges are not unique to HLDs but are shared by populations with neuromuscular or myelopathic impairment undergoing spinal deformity surgery. In scoliosis associated with cerebral palsy, the absence of baseline signals is well documented and correlates with clinical severity and short stature [34]. Generally, in neuromuscular scoliosis, the absence of a baseline is a widely recognized challenge, with reduced monitorability even when technical optimization is applied [27]. In spinal muscular atrophy (SMA), studies show that MEPs and SSEPs can often be obtained with extreme difficulty and lower amplitude responses requiring combined recording from multiple lower‐limb muscles and parameter adjustment [35]. In severe myelopathy, multimodal monitoring is essential, and signal loss during spinal cord manipulation serves as a highly predictive warning, even though compromised baselines reduce sensitivity [36]. In hereditary spastic paraplegia, axonal degeneration of the corticospinal tract, analogous to the secondary degeneration seen in leukodystrophies, impairs motor conduction and may limit the acquisition of MEPs [37, 38]. Collectively, these populations illustrate a common principle: Intraoperative monitoring in neuromuscular and myelopathic disorders requires realistic expectations regarding monitorability, parameter optimization, and the integration of multiple modalities rather than exclusive reliance on a single signal.

This case report highlights the unique challenges of IONM in patients with *TUBB4A*‐related HLD undergoing scoliosis correction. We demonstrate that, although MEPs and SSEPs may be absent due to the pathophysiology of the disease, other modalities such as free‐running EMG and the BCR can offer reliable monitoring of sacral spinal cord integrity. This case underscores the need for adapted IONM protocols and a multimodal approach for patients with pre‐existing neurological conditions, filling a critical gap in the literature and contributing to clinical practice in this rare and complex population (Figure 6).

**FIGURE 6:**
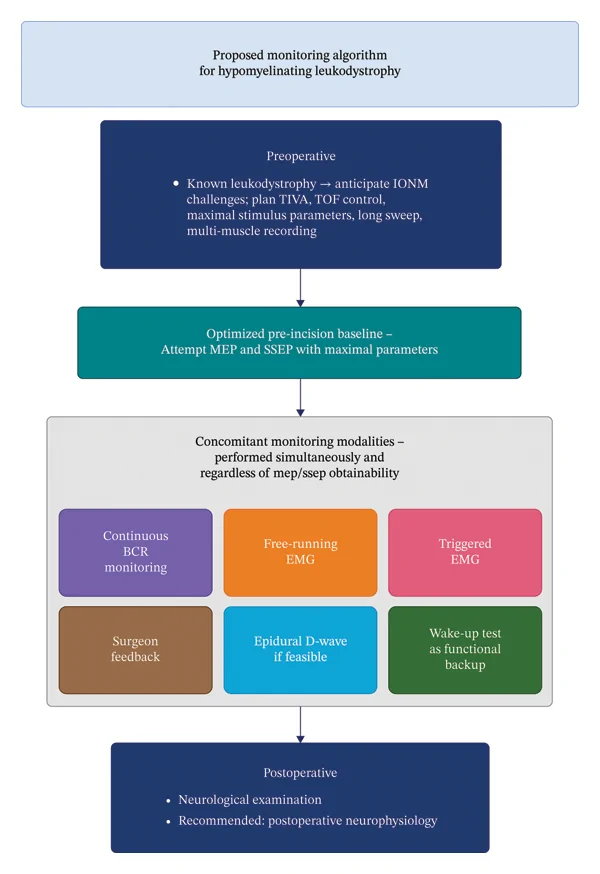
Proposed algorithm for intraoperative monitoring in hypomyelinating leukodystrophy. The flowchart illustrates the adaptive multimodal strategy: preoperative planning, acquisition of an optimized MEP/SSEP baseline, and the concomitant and mandatory execution of additional modalities followed by postoperative neurological and neurophysiological assessment. BCR, bulbocavernosus reflex; EMG, electromyography; MEP, motor‐evoked potential; SSEP, somatosensory evoked potential; TIVA, total intravenous anesthesia; TOF, train‐of‐four. This figure was generated with the aid of artificial intelligence (GPT‐Image‐2, OpenAI) based on a schematic and parameters provided by the authors, who reviewed and validated the content and assume responsibility for the figure.

This report has a few limitations. First, as a single‐case report, it precludes generalizability to the broader population of patients with leukodystrophies. Second, the absence of a preoperative MEPs study made it impossible to characterize the baseline functional status of the corticospinal tract prior to surgery. Third, the lack of postoperative neurophysiological assessment prevents confirmation of whether nerve conduction was maintained or recovered over time. Fourth, functional outcomes were assessed qualitatively without the use of validated quantitative instruments for motor function, sphincter function, or quality of life. Finally, the 4‐month follow‐up period does not allow for the assessment of long‐term outcomes. These limitations constrain any definitive interpretation of IONM efficacy in this context, highlighting the need for future studies involving larger cohorts, standardized pre‐ and postoperative neurophysiological protocols, and extended clinical follow‐up.

Nevertheless, some lessons learned from this case include conducting a thorough medical history and physical examination is fundamental for identifying potential pre‐existing and unreported deficits, as well as informing the patient and their caregivers about the benefits and limitations of intraoperative monitoring; meticulously analyzing previous examinations and requesting multimodal preoperative evoked potential testing; discussing the approach, risks, and potential outcomes with the surgical team beforehand; performing multimodal neuromuscular electrical stimulation, including SSEPs and MEPs, and evaluating the acquisition of additional reflexes that help assess motor pathways (BCR and H‐reflex); and maintaining clear and effective communication with the surgical team, and promptly reporting abnormal responses for possible adjustments to the surgical strategy.

## Author Contributions

Marta Rodrigues de Carvalho: conceptualization and design of the work, data acquisition, analysis, interpretation, and writing of the manuscript; Eduardo Henrique Chiovato Abdala: data acquisition and writing of the manuscript; Carlos Fernando Pereira da Silva Herrero, Vitor Marques Caldas, and Narcélio Mendes Ferreira Filho: analysis and interpretation of data and review of the manuscript.

## Funding

No specific grant from funding agencies in the public, commercial, or not‐for‐profit sectors was received.

## Disclosure

All authors have approved the final version of the manuscript and agreed to be responsible for all aspects of the work.

## Consent

Informed consent was obtained from the legal guardian of the participant included in this study. The CARE guidelines (for CAse REports) were followed carefully.

## Conflicts of Interest

The authors declare no conflicts of interest.

## Data Availability Statement

The data that support the findings of this study are available on request from the corresponding author.
